# DeepBrain: Experimental Evaluation of Cloud-Based Computation Offloading and Edge Computing in the Internet-of-Drones for Deep Learning Applications

**DOI:** 10.3390/s20185240

**Published:** 2020-09-14

**Authors:** Anis Koubaa, Adel Ammar, Mahmoud Alahdab, Anas Kanhouch, Ahmad Taher Azar

**Affiliations:** 1 Department of Computer Science, College of Computer & Information Sciences, Prince Sultan University, Riyadh 11586, Saudi Arabia; abu.msab96@gmail.com (M.A.); Akanhouch@psu.edu.sa (A.K.); aazar@psu.edu.sa or ahmad.azar@fci.bu.edu.eg (A.T.A.); 2 CISTER Research Centre, ISEP, Polytechnic Institute of Porto, 4200-465 Porto, Portugal; 3 Faculty of Computers and Artificial Intelligence, Benha University, Banha 13511, Egypt

**Keywords:** unmanned aerial vehicles (UAVs), deep learning, cloud computing, smart cities, Internet-of-Things, remote sensing

## Abstract

Unmanned Aerial Vehicles (UAVs) have been very effective in collecting aerial images data for various Internet-of-Things (IoT)/smart cities applications such as search and rescue, surveillance, vehicle detection, counting, intelligent transportation systems, to name a few. However, the real-time processing of collected data on edge in the context of the Internet-of-Drones remains an open challenge because UAVs have limited energy capabilities, while computer vision techniquesconsume excessive energy and require abundant resources. This fact is even more critical when deep learning algorithms, such as convolutional neural networks (CNNs), are used for classification and detection. In this paper, we first propose a system architecture of computation offloading for Internet-connected drones. Then, we conduct a comprehensive experimental study to evaluate the performance in terms of energy, bandwidth, and delay of the cloud computation offloading approach versus the edge computing approach of deep learning applications in the context of UAVs. In particular, we investigate the tradeoff between the communication cost and the computation of the two candidate approaches experimentally. The main results demonstrate that the computation offloading approach allows us to provide much higher throughput (i.e., frames per second) as compared to the edge computing approach, despite the larger communication delays.

## 1. Introduction

Artificial Intelligence (AI), in particular, deep learning, and Unmanned Aerial Systems (UAS) are the two most prominent technologies in the last five years [1]. The Business Intelligence and Strategy (BIS) research reported that the total revenue of UAS in 2018 was worth USD 785.8 Million, and is expected to reach by 2024 the value of USD 1.97 Billion [2]. The Compound Annual Growth Rate of drones’ market is predicted to be 16.83% in the next five years [3]. On the other hand, Markets and Markets Research Private reported that the market of deep learning in 2017 was valued to USD 2.28 Billion, and is projected to reach 18.16 Billion by 2023 with a Compound Annual Growth Rate of 41.7% [3].

While the main objective of drones is to collect visual data including aerial images and videos (in addition to other types of data), deep learning algorithms are nowadays the de facto standard for processing images and extracting useful data using different techniques such as classification, object detection, semantic segmentation, and instance segmentation [4]. Several recent research works have leveraged deep learning algorithms based on convolutional neural networks to process aerial images collected from drones [5,6,7,8,9]. In [5,6], the authors conducted a comparative study between the two state-of-the-art algorithms YOLOv3 and Faster Region-CNN (RCNN) for the detection of cars from aerial images. In [7], the authors proposed a technique using Generative Adversarial Networks (GANs) for domain adaptation to improve the semantic pixel-level segmentation of aerial images of urban environments with different characteristics. Ammour et al. in [8] developed a pre-trained CNN in addition to a support vector machine (SVM) classifier for the detection and counting of vehicles from high-resolution drone images. In [9], the authors investigated the problem of vehicle tracking from aerial images. The authors in [10] proposed CNN architectures to automate the classification of aerial scenes of disaster events, such as fires, earthquakes, floods, and accidents. The works mentioned above demonstrate the recent trend in coupling UAS applications with deep learning algorithms. However, these works are mostly based on the offline analysis of aerial images collected from drones, and none of them have considered the processing of images or videos in real-time, meaning, as soon as they are collected from the drone.

### 1.1. Problem Statement

The processing of UAV images in real-time can be achieved in two different ways: (i.) using onboard processing of images with a GPU board (e.g., Nvidia Jetson TX2 Board) (ii.) using computation offloading by migrating the computation of deep learning algorithms from the drone to the cloud. The second approach is an emerging trend since 2010, under the context of cloud robotics [11,12]. The idea consists of leveraging cloud resources to improve the performance of robotics applications for computation-intensive tasks. Each approach has its advantages and drawbacks. On the one hand, computation offloading reduces the load on the drone/robot by offloading computation to the cloud, and as a consequence, reduces the energy consumption related to computation. On the other hand, it requires high communication bandwidth for high-speed transmission of images/video frames to the cloud, which may affect the quality of service of the application in terms of reliability and real-time guarantees [13]. Furthermore, the gain of energy saving from a computation perspective is compromised by extra energy dissipation due to communication. This topic is a typical research problem pertaining to adopting optimal computation offloading strategies in cloud robotics that were addressed with different approaches in recent works, including Game Theory [14,15], Markov Decision Processes [16,17], and computational intelligence [18].

### 1.2. Main Contribution

In this paper, we consider the problem of computation offloading of aerial images and videos from drones to the GPU-enabled cloud to process aerial images using deep learning algorithms in real-time, and provide timely feedback on the observed scene to the end-users. The contributions of the paper are summarized as follows:

- First, we propose, DeepBrain, a cloud-based Internet-of-Drones architecture that provides users with seamless access to drones over the Internet, and allows drones to offload deep learning computation to the cloud for real-time processing of the collected visual data.
- Second, we present an experimental study that demonstrates the feasibility of the proposed architecture, and we evaluate the performance of the computation offloading approach and edge computing approach in terms of energy consumption, cloud server utilization, and real-time guarantees. We also compare the computation offloading approach and the onboard computation approach. DeepBrain demos are available in [19].

The remainder of this paper is organized as follows. In Section 2, we discuss the recent works on computation offloading for cloud robotics. In Section 3, we describe the DeepBrain system architecture. In Section 4, we present the experimental study and performance evaluation of the proposed architecture. Section 5 concludes the paper and outlines future works.

## 2. Related Work

Table 1 summarizes the most recent trends in computation offloading related to the present work. In [20], the authors developed Neurosurgeon, a program that can automatically divide Deep Neural Networks (DNN) into neural layer granularity between mobile devices and the cloud. The neurosurgeon program can be used for varying latency and the highest mobile energy consumption rates in DNN systems and hardware platforms, wireless communications and server loads.

In [24], Chaari et al. proposed a distributed cloud robotics framework for computation offloading, based on Kafka platform. They concluded that computation offloading reduces robot central processing unit (CPU) load but increases application execution times due to delays. In [25], the authors have proposed a heuristic technique for efficient computation offloading of deep learning applications from mobile devices to the cloud using 5G networks. They proposed a framework for appropriate offloading strategies using edge computing for a centralized unit (CU)-distributed unit (DU) architecture. They evaluated the performance of their approach using simulations, and they showed that they could shorten the delays for deep learning tasks.

In [17], the authors addressed the problem of optimal computation offloading of adhoc mobile applications to the cloud using cellular networks. They have used deep reinforcement learning to reach optimal offloading decisions. The authors have considered several parameters, including the uncertainty of users’ motions and the availability of cloudlet resources. The Markov decision process modeling is used to formulate the offloading problem, and the objective is to optimally allocate tasks that should be processed locally by the users and those that must be offloaded to cloudlet while maximizing the utility function and minimizing the energy consumption, task processing delays, task loss probability, and payment. Finally, the deep Q-network is used to learn the best decision for the Markov decision process. The results were evaluated using simulation, and the effectiveness of the proposed approach is demonstrated.

In [22], the authors developed optimal joint connectivity, storage, and computing resource management system for vehicular networks. The deep reinforcement learning approach with the multi-time scale framework is designed to optimize resource allocation.

In [26], the authors applied computation offloading to vehicular networks. They started from the observation that the in-vehicle computing resources are limited and mentioned the benefit for vehicular networks to leverage the computing and storage capabilities of edge and cloud computing to improve their performance. They formulated the problem as a resource scheduling problem and aimed to optimize multiple objective functions and constraints. The contribution of that paper consists of the proposal of a knowledge-driven computation offloading framework using deep reinforcement learning. The benefit of this approach is that the framework learns online from the vehicular services as they execute and adapt the offloading tasks based on the knowledge gained in the past.

In [27], the authors suggested offloading approach based on the deep learning response time. It supports edge/cloud. This decides reliably whether the neighbor node, edge/fog node or cloud node can be offloaded.

In [28], the authors proposed a near-end network solution for mobile edge/fog offloading using the deep Q-learning framework. The results indicate that the proposed offloading performs better for time and latency execution and energy consumption.

The authors in [29] presented the Nonorthogonal Multiple Access (NOMA) system for mobile edge computing (MEC) vehicular network, which reflects an early attempt to offload network traffic by using spectrum reuse and high-efficiency computing technologies in an exhaustive manner.

In [23], the authors presented a mobile edge system and optimized deep reinforcement learning methods for mobile edge computing as well as storage and communication with the mobile device. Different scenarios were evaluated concerning the edge caching and device offloading in mobile edge systems. The authors designed a framework that achieved near-optimal performance.

In [35], the authors addressed the problem of computation offloading for mobile devices in multi-access edge computing networks. The main contribution of that work is developing collaborative offloading approaches that use data caching and social relationships to reduce the delays and optimize energy. The performance of the proposed method was demonstrated using simulation. In [16], the authors proposed a computation offloading framework for IoT applications, particularly tailored for remote areas that lack edge and cloud infrastructure. Their approach consists of using unmanned aerial vehicles as edge devices to provide access to cloud services. They also formulated the computation offloading problem as a Markov decision process and used deep reinforcement learning to determine the optimal solution to the problem. They have also used network virtualization for the dynamic allocation of resources on the edge server. They have evaluated the performance of their approach using simulation, and they demonstrated that the DRL approach allows providing the lowest total cost in terms of delay, execution time, energy efficiency as compared to the greedy approach.

In [21], the authors made a demonstration on the effect of the network conditions for the remote control of drones over cloud infrastructure. They have developed a prototype to investigate the quality of experience for controlling the drone over the cloud. In [36], the authors investigated the use of cloud service to extend the flying time of a team of drones. The contribution consists of the proposal of an adaptive task scheduler to offload computation to the cloud to achieve faster execution times and reduce the drone’s energy consumption. The paper also investigated the offloading of computation from one drone to another. They validated their approach by conducting experiments on a Raspberry PI to emulate a drone and evaluated the energy consumption with and without computation offloading for video streaming applications.

In [37,38], the authors have proposed Dronemap Planner, a cloud-based management system for the Internet-of-Drones. The objective was to provide a software interface for the access and management of drones over the Internet. However, the authors did not address the issue of computation offloading.

In [31], the authors suggested the UAV edge computing network’s intelligent Task Offloading Algorithm (iTOA). Compared to current procedures, iTOA can intelligently perceive the network environment by determining the download operation based on a profound Monte Calor Tree Search (MCTS). The authors also proposed the Split Deep Neural Network (SDNN) to speed up the search convergence for MCTS, to provide the prior MCTS likelihood. The proposed iTOA increases service latency efficiency by 33% and 60% compared to game theory and greedy search methods.

In [30], the authors developed a deep-reinforcement-learning-based framework for 5G-enabled vehicle networks using licensed cell spectrum together with unlicensed channels. The proposed framework achieved an overall better offloading cost compared to Q-learning and full cellular offloading algorithms.

In [32], the authors discussed the limitations in data transfer, limited energy, and inadequate resources of the current Mobile Edge Computing (MEC) network, and developed a new MEC architecture solution. As a relay edge computing node and UAV powered MEC networks, unmanned aerial vehicles (UAV) have been added. By combining the UAV location optimization algorithm with the Long Short Term Memory (LSTM) based task prediction algorithm, an energy efficiency optimization algorithm is proposed based on a three-layer computation offloading strategy. The test results show and confirm that the energy consumption of UAV is reduced efficiently, and the proposed algorithm is used to dynamically schedule the task offloading strategy.

In [33], a new framework of task scheduling is presented in the unmanned aerial vehicle-aided mobile edge computing (UMEC). The agent-enabled task offloading architecture is provided. The agent offloads strategies and helps carry out task offloading by integrating resources into the user devices, UAV, and edging cloud. The test results showed that the implementation of the agent in computing tasks would reduce delays and energy consumption significantly.

In [34], the authors considered the issue of implementing an efficient method for processing the UAV-collected data involving computer offload-sharing decision-making problems in a multi UAVs-aided traffic management scenario. The fundamental goal was to reduce computing time while reducing the operating energy and the expense of measuring and communicating. First, the authors suggested a new device architecture for offloading and exchanging computations. A new device utility function is then developed, which combines calculation time, overhead energy, link quality, communications, and computing costs. The results showed that the proposed game-based model outperforms other approaches by providing higher output in the overall system utilities and provides a more efficient time and energy average for data processing which ranges from 43 % to 97 % according to the calculation approach.

In [39], an analysis of computational offloading mechanisms based on machine learning (ML) in the mobile edge computing (MEC) environment is discussed in the form of a classical taxonomy for the determination and the presentation of contemporary mechanisms on this critical subject.

In [40], the authors presented a detailed review and recent progress on the offload modeling of edge computing. Furthermore, they highlighted and addressed some recommendations and problems for edge computing offloading modeling in research fields.

In this paper, we investigate experimentally two computation approaches used in the Internet-of-Drones, namely the cloud-based computation offloading and edge computing for the Internet-of-Drones. We first design a system architecture for the Internet-of-Drones for computation offloading of deep learning applications. Then, we evaluate its performance as compared to edge computing (computation onboard of the drone) in terms of energy, delay, and bandwidth. This study’s added value is to provide a quantitative evaluation of the two computations approaches based on real-world experiments, in contrast to previous works mostly based on simulation.

## 3. Proposed System: Deepbrain System

### 3.1. The Design Requirements

The objective of the DeepBrain system is to provide a solution to the computation offloading of heavy and computation-intensive tasks/applications from a small unmanned aerial system to the cloud system to (1) reduce energy consumption and thus extend the mission lifetime of the UAV, (2) to leverage the use of cloud resources for the execution of deep learning applications.

#### 3.1.1. Functional Requirements

In terms of functional requirements, the UAV should satisfy the following:

- Be able to execute a mission where it collects aerial images of points of interest using a high-resolution camera.
- Images are then transmitted to the cloud system through cellular technology (i.e., 4G/5G) for visual recognition and identification of objects and events.
- The cloud should have sufficient resources of GPU instances to run deep learning algorithms on the collected images.
- The results are shown to the end-users through a monitoring dashboard, and control commands or actions are sent back to the drone.

#### 3.1.2. Non-Functional Requirements

Regarding non-functional requirements, the UAV shall satisfy the following requirements:

- Real-time: the delay from the instant when the image is taken until the result or action is displayed to the end-user must be short and bounded. The maximum end-to-end delay shall not exceed 500 ms for the appropriate performance of the system. Such a real-time guarantee can be provided through typical 5G networks offering network bandwidth in the order of hundreds of Gigabytes and low latancies below 10 ms.
- Scalability: the DeepBrain system shall support a large number of UAVs simultaneously at the scale of a city. Several hundreds of UAVs shall have accessibility to the system for performing various missions. Distributed load balancing mechanisms should be implemented to avoid overusing particular resources.
- Energy-Efficiency: DeepBrain shall provide efficient energy management to extend the operational lifetime of the drone. Effective management between computation and communication has to be achieved. Besides, the energy consumption at the cloud side shall also be controlled for green cloud computing purposes.
- Security: DeepBrain shall provide secure access to UAVs and users and shall be able to detect and prevent malicious attacks on the system.
- Safety: The system shall ensure the safety of operations of drones and implement failsafe strategies when any hazardous event happens (e.g., communication loss with the cloud, GPS loss, occlusions)
- Reliability: The system shall perform correctly during its operation and provide a fault-tolerance mechanism to recover from any unexpected situation.

### 3.2. Motivating Scenario

In this section, we present a scenario that motivates the need for the DeepBrain system. Let us consider multiple UAVs in a city, located at different positions and may be requested at any time to perform a data collection mission.

In the traditional UAV missions, the pilot has to go on-site with the drone where the operation happens and collect data according to Part 107 regulations. The main regulations under Part 107 are: (1) mission must be executed during day times; (2) only visual line-of-sight missions are allowed; (3) two pilots must be present at the site of operation, while the second pilot acts as a visual observer; (4) operations over a densely populated area and certain airspaces are not allowed; (5) multiple drones’ operations in the same space are not allowed.

In traditional drone operations, the manual drone pilot makes mission operations tedious and at risk because it is controlled by error-prone humans. The integration of drones over the Internet alleviates the need for human operators’ intervention as the control is made more automated through smart controllers over the cloud, which may relax some of the constraints imposed by Part 107 regulations such as daytime operation and visual line of sight.

On the other hand, in traditional drone operations like in photogrammetry applications, data (i.e., image) is collected and stored in the drone. Then, it is offloaded after the mission in a smart device or a computer for further processing, using deep learning frameworks or photogrammetry software. In this way, it is not possible to perform real-time processing of images collected from the drone, which is needed in several mission-critical applications such as search-and-rescue and disaster management. Using GPU-based onboard computers on the drones does not substantially solve the problem because GPUs dramatically consume tremendous energy, which leads to reducing the flight time. The current flight time of typical small UAVs is between 30 and 60 min for just flying without any extra computation load.

The limitations mentioned above are the driving motivation towards the development of a cloud-based computation offloading framework for small UAVs for deep learning applications. In the next section, the DeepBrain system is presented.

### 3.3. The Deepbrain Architecture

**Figure 1:**
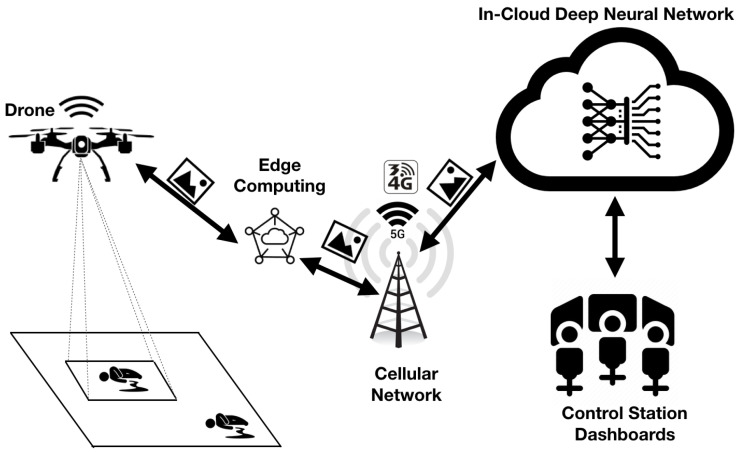
DeepBrain architecture.

In this section, we present the system architecture of DeepBrain, which is depicted in Figure 1.

The DeepBrain system is composed of four layers (or subsystems):

- The Unmanned System Layer: it represents the UAV subsystem that is responsible for aerial image collection from the monitored site. It has to be noted that the unmanned system can also be a ground robot as the principle applies in the same way for any unmanned system (aerial, ground, or even submarine). Without loss of generality, in this paper, aerial systems have been considered. The UAV is equipped with sensing capabilities, namely a high-resolution camera to capture images. It also has a wireless communication interface to communicate with the edge/cloud servers. Drones connected through the cellular (4G/5G) networks have been considered. A typical setup would be to use a WiFi interface on the drone to connect to a 4G/5G portable WiFi router.
- The Edge Layer: This layer aims at increasing the scalability of the system and reducing the load of the cloud layer. Edge computing centers differ from the cloud as they are located closer to the end-devices (i.e., drones in the proposed case) and increase the decentralization of computing among multiple servers rather than on a single server. In the DeepBrain system, the Edge servers help to migrate some of the deep learning computations from the cloud to the edge. In fact, with hundreds or thousands of drones collecting images and sending them to one cloud server to process, the latter’s load might not be able to scale-up with the increasing intensive computation demands. Thus, edge computing is much more effective for real-time video stream processing and responsive feedback control of the drone.
- The Cloud Layer: The cloud subsystem deploys deep neural network algorithms that require extensive computing and storage resources that cannot be handled by edge servers. Considering the abundant resources of the cloud, it is used for processing images/videos, which requires additional resources as compared to the edge capabilities. The usage of the cloud is more suitable for less time-critical applications but has more stringent requirements in terms of computing and storage. The cloud server may also provide services not provided in the edge layer. For instance, the cloud server may deploy generative adversarial networks (GANs) and semantic segmentation algorithms [7], which are known to be more computation-intensive than standard classification and object detection deep learning applications. The cloud also offers all the drones and users management capabilities to ensure their connectivity, communication, authentication, and the availability of the services. We have already implemented this functionality in the Dronemap Planner system [37,38,41].
- The End-User Layer: This layer represents the end-users who are using the DeepBrain system through the Internet. They interact with the cloud through Web services Application Program Interfaces (APIs). They use interactive dashboards to monitor the states of their drones in real-time and to send appropriate commands when needed. They also receive the real-time video stream broadcasted from their drones after being processed by deep learning applications either located at the edge or on the cloud. The end-users may define the required business rules for their applications, such as geofencing, unmanned traffic management, path planning requirements, through their command dashboards.

DeepBrain architecture has several advantages. First, it eliminates the need for the pilot to be in communication range of the drone during the mission. Second, it allows a scalable computation offloading to the edge/cloud to promote deep learning applications for low-cost UAVs. Third, it leverages the edge/cloud resources for the real-time processing and storage of video streams broadcast by the drones. In this next section, we present an experimental proof-of-concept of the deep brain architecture, and we evaluate the performance of the DeepBrain system for different use cases.

### 3.4. System Components

**Figure 2:**
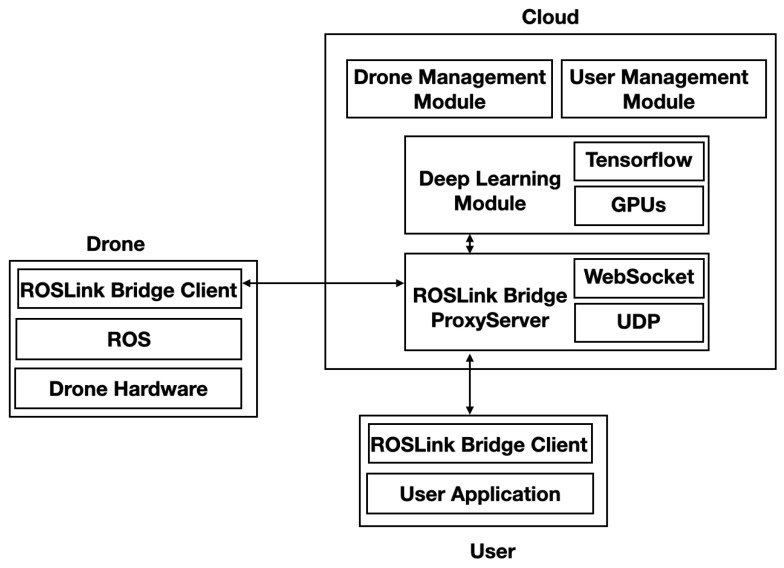
DeepBrain system components.

DeepBrain system components are illustrated in Figure 2. It consists of three main subsystems: (1) drone subsystem, (2) cloud subsystem, and (3) end-user subsystem.

#### 3.4.1. Drone Subsystem

It consists of the drone hardware, including motors and sensors (e.g., GPS, Compass, Accelerometer, Gyroscope) and their low-level drivers. The drone uses the Robot Operating System (ROS) [42] as a middleware between the hardware and high-level applications. ROS allows us to interact with all the drones’ sensors and actuators easily. It also enables making abstraction of the hardware resources through software APIs. On top of ROS, we use the ROSLink Bridge Client to transfer any data collected from the drone through ROS to the cloud using the ROSLink protocol [43]. It is a protocol that we developed to ensure the communication between unmanned systems (drones/robots) with any cloud server. The idea of ROSLink is to extract data from ROS (e.g., images, sensor status, position, motion state), embed them into JSON-serialized messages, and send them to the cloud.

#### 3.4.2. Cloud Subsystem

It represents a remote server with extensive resources in terms of computation, storage, and networking. It acts as a proxy between the drones and the end-users. The cloud subsystem is responsible for the management of drones and users active in the system. It keeps track of every drone/user and ensures the communication between them in real-time. It performs this through the ROSLink Bridge Proxy Server using the ROSLink protocol to interact between the ROSLink clients of the drones and users. The ROSLink protocol supports two communication interfaces, namely UDP for best-effort traffic, and Websockets for more reliable and real-time communication. Apart from communication, the DeepBrain cloud subsystem provides a deep learning module for the performing inference on images collected by drones and received by the cloud. This module leverages the cloud server’s multi-GPU capabilities to speed-up the inference process of multiple images from different drones. For example, our cloud server has 8 × RTX 8000 GPUs, each offering 48 GB of RAM. Embedded devices cannot provide these GPUs capabilities in the drone, which justifies the need for computation offloading of some computation-intensive tasks when local processing is not sufficient. In this paper, we will investigate experimentally the tradeoff between the cost of communication and computation for two different strategies, namely with and without computation offloading.

#### 3.4.3. User Subsystem

The user subsystem represents the end-user application that interacts with the drone through the cloud. It also uses the ROSLink Bridge Client to communicate with the ROSLink Proxy of the cloud subsystem. The user application consists of a dashboard that allows us to monitor the drone’s activity in real-time. It also visualizes the results of deep learning inference made on the cloud. The user application can also send a command to control the drone remotely through the Internet. Considering delay and jitters, the user uploads pre-defined missions as waypoints and actions that the drone executes autonomously. Teleoperation using a joystick is also possible.

### 3.5. Computation Approaches

**Figure 3:**
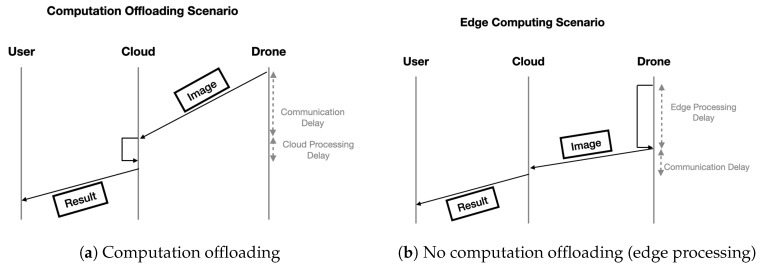
Computation offloading

In this study, we consider two computation approaches, as illustrated in Figure 3:

- Computation offloading (Figure 3a): it refers to the case when deep learning computation is completely offloaded to the cloud using video streaming.
- Edge computing (Figure 3b): It refers to the case when deep learning computations are performed at the edge (i.e., the drone) using devices with embedded GPUs (e.g., Jetson Nano, Raspberry Pi).

In this paper, we will experimentally evaluate the benefits and limitations of each approach and the tradeoff in terms of communication cost and computation cost. In edge computing, it can be observed in Figure 3b that the cost of computation is typically high in the edge compared to the communication cost. The reason is that edge computing typically uses CPU or low-cost embedded GPUs to process images with deep learning inference models (e.g., object detection), then sends only the results of the inference to the cloud. In such a case, the processing time is typically more significant than communication delay. On the other hand, in the case of computation offloading, as illustrated in Figure 3a, the communication cost dominates the cloud processing cost since messages exchanged between the drone and the cloud will carry image data, which typically has a much larger size and consumed greater bandwidth. On the other hand, the processing delay should be smaller because of the GPUs’ high-performance capabilities on the cloud side. Image processing techniques can also reduce the communication delay’s impact, but at the expense of lower inference accuracy due to lower resolution images. This paper will analyze the communication and computation costs of the two approaches and investigate the different tradeoffs experimentally.

## 4. Results and Experimental Analysis

In this section, we present an experimental study of the DeepBrain system to demonstrate its feasibility and effectiveness. The main objective of this study is to assess the impact of the computation offloading of deep learning applications on the drone’s energy consumption, network bandwidth, and real-time guarantees.

### 4.1. Experimental Setup

We have built an experimental testbed of 4G connected drones to evaluate the performance of the DeepBrain system under different conditions. Experimental field demos of the DeepBrain system are available in [19].

#### 4.1.1. 4G Custom Drones

**Figure 4:**
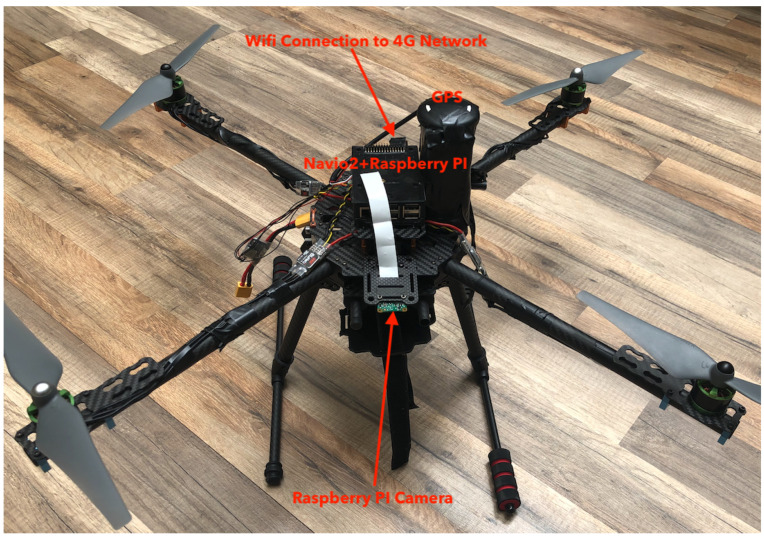
4G custom drones.

In the experiments, we have used our custom-made drones that we specifically built to illustrate the concept of 4G connected drones and the computation offloading from the drone to the cloud of deep learning computer vision applications. Figure 4 shows the 4G custom drone that we have deployed in the football field of Prince Sultan University. We built two quadcopters with a frame of 250 mm and one drone with a frame of 450 mm. Every drone has a Navio2 autopilot shield on a Raspberry PI 3 onboard computer. The drones are equipped with a USB camera connected to the Raspberry PI 3. We use the ROSLink protocol [43] to broadcast the internal state of the drone using the mavros interface, and also to broadcast the video stream.

#### 4.1.2. Cloud Server and GPU Server

In our experimental work, we used a DreamCompute Cloud instance (gp1.hyperspeed flavor, 16 GB Ram, 8 Virtual CPUs, 80 GB Hard drive, Ubuntu 16.04) from DreamHost service provider to run the Dronemap Planner [37], which acts as a relay between the drone and the GPU server. The latter is responsible for the execution of the deep learning computer vision algorithms for object detection and geolocation of the detected objects. We used the GPU server as a GPU-enabled cloud computing service that provides object detection services through the Dronemap Planner cloud server. The GPU machine has an NVIDIA GeForce GTX 1080 Graphics card with 8 GB of RAM, and a processor Intel Core i7-8700K at 3.7 GHz.

#### 4.1.3. Experimental Scenarios

We evaluate the performance of DeepBrain in terms of three performance metrics, namely:

- **Scenario 1:** Energy consumption (i.e., mission lifetime). In this scenario, we compare the energy consumption of the drone when running the deep learning algorithm on the drone itself against the scenario when running it on the cloud.
- **Scenario 2:** Cloud Server Utilization. In this scenario, we evaluate the utilization of the cloud server both in terms of bandwidth usage with and without computation offloading.
- **Scenario 3:** Real-time guarantees. We evaluate the response time of the DeepBrain architecture with and without computation offloading.

### 4.2. Results

#### 4.2.1. Impact on UAV Energy

In this scenario, we considered two cases. In the first case (with computation offloading), a drone streams video frames from the Raspberry Pi to the cloud using the ROSLink protocol to be processed remotely on the GPU server. In the second case (without computation offloading, using onboard processing), the drone has an embedded NVIDIA Jetson TX2 Board, as an onboard GPU machine, to process the collected video frames in real-time without computation offloading. We used different 3S and 4S LiPo batteries to power the Raspberry Pi and NVIDIA Jetson TX2 Board. We measured the consumed energy for both scenarios to evaluate the energy efficiency of the computation offloading method. Table 2 shows the average voltage decrease rate (Volt Per Second) with and without computation offloading for five experiments. It also shows the instant power consumption in Watt measured using a Power Meter on a Raspberry PI with and without running the MobileNet-SSD [44] object detection algorithm onboard using Tensorflow Lite.

In comparison, we consider the case of non-flying drones (just streaming data and videos), to focus only on the impact of communication and processing without the effect of motors on energy. We observe that computation offloading enables us to save energy as compared to onboard GPU processing because the voltage decrease rate and also power consumption in watt in the case of computation offloading is almost half of that of the onboard GPU processing. This allows extending the mission flight time of the drone when the extensive computation is offloaded to the cloud. When the drone is flying, the impact of motors on energy consumption is around 40 times higher than the energy consumed by computation and communication.

#### 4.2.2. Impact on Bandwidth

In this section, we evaluate the impact of the computation offloading of computer vision processing to the cloud on the consumed bandwidth (i.e., throughput). We run experiments with one to five drones, and we observed the impact on the throughput. We considered two cases: In the first case, the drones offload video to the cloud. In the second case, the drones do not perform computation offloading and process the video locally.

**Figure 5:**
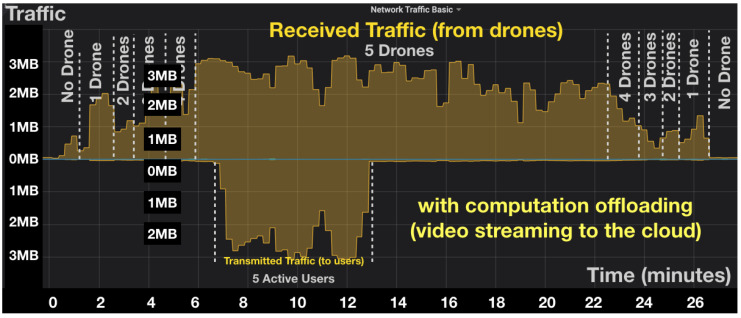
In/out throughput in the cloud server with computation offloading.

**Figure 6:**
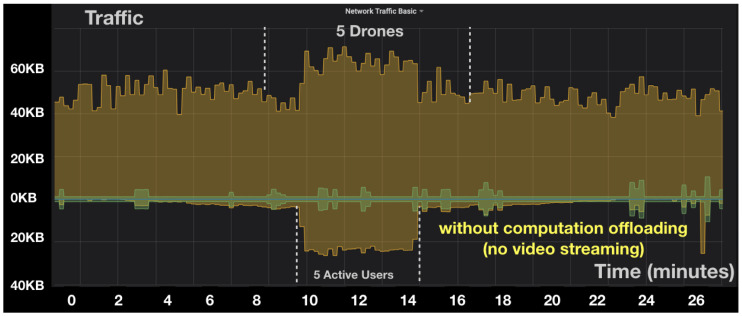
In/out throughput in the cloud server without computation offloading.

**Figure 7:**
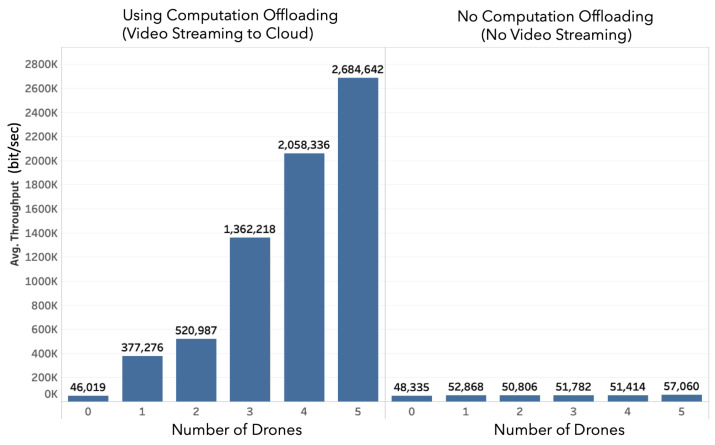
In-cloud average throughput vs. number of drones.

Figure 5 and Figure 6 show the received and transmitted traffic over time for a different number of drones measured at the cloud level, in case of computation offloading and without computation offloading, respectively. It can be observed that when increasing the number of drones, the received traffic increases linearly. The output of traffic increases with the number of active users connected to the drones to monitor and control them. Regarding the comparison between computation offloading and local processing approaches, it is clear that offloading the video stream to the cloud induces a much higher bandwidth and resource utilization at the cloud level. The observation is confirmed from a statistical perspective in Figure 7. The figure presents the average received bandwidth as the number of drones increases, with and without computation offloading. From Figure 7, the bandwidth increases almost linearly with the number of drones because, in our scenario, each drone is streaming a constant-bit rate video stream, thus, the total bandwidth is linearly cumulative with respect to the number of drones. It can be noted that the computation offloading approach requires up to 32 times more bandwidth resources in the cloud as compared to a local processing approach. While this is acceptable for our case of five drones, it might become an issue if the number of drones scales larger. It is therefore important to make an efficient balance between computation offloading and local processing at the edge to avoid excessive usage of the cloud network resources.

**Figure 8:**
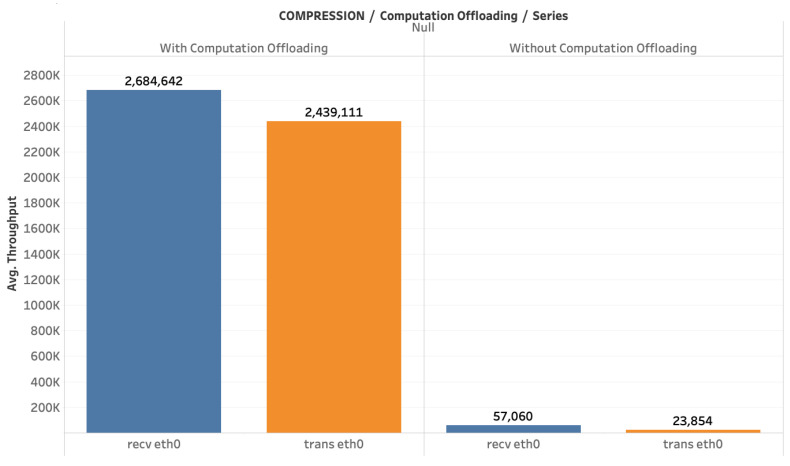
Throughput comparison, with and without computation offloading.

Figure 8 depicts the average throughput with and without computation offloading for both traffic received and sent by the cloud, during the period of having five active drones. The results are consistent with those of Figure 7. Besides, we observe that the traffic forwarded by the cloud is 10% less than the traffic received. This is mainly due to the latency and jitter induced by the network. In the case of local processing, average throughput is 50 times less than in the case of computation offloading, for five drones under the considered configuration. This result demonstrates the demanding requirements in terms of bandwidth for computation offloading of video frames from drones to the cloud.

#### 4.2.3. Real-Time Guarantee

In this section, we focus on the evaluation of the real-time metrics for the deepbrain system to assess the impact of computation offloading on the real-time quality-of-service. In particular, we observe and measure the following timing metrics:

- The Cloud Execution Times: it represents the time from the instant when a packet is received by the cloud from the drone until the instant when the packet is forwarded from the cloud to the end-user.
- End-to-End Network Delays: it represents the delay for a message to be transmitted from the drone to the end-user through the cloud. We estimate this time by computing the round-trip time for any message sent by the drone divided by two. The reason is to compute the delay using one reference clock to avoid the effect of clock skewing of two distinct machines. In fact, measuring the delay with different clocks would add much more complexity and uncertainty in the computation of delays, and would require accurate clock synchronization, which is challenging to achieve.
- Frames Per Second (FPS): it measures the number of frames per second received by the end-user. It is measured with respect to a window of 10 frames received at the destination.

##### Execution Time

**Figure 9:**
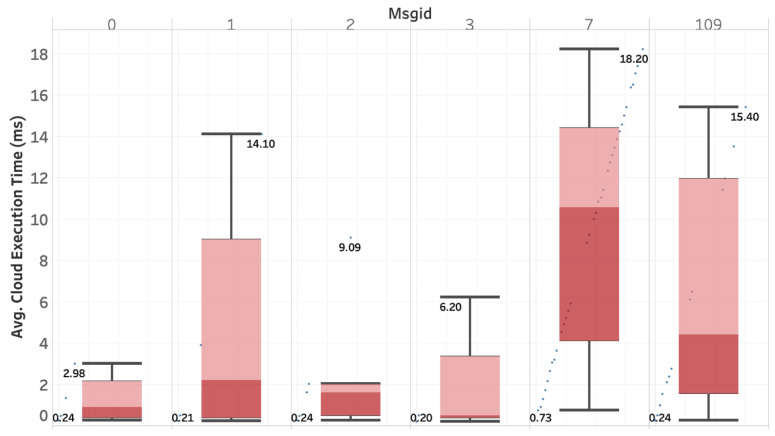
Cloud execution time per message type.

The cloud execution time is presented in Figure 9 for one drone streaming its video to the cloud. The figure presents the execution times of messages with different types (i.e., each type has a specific message ID). The message ID 0 refers to heartbeat messages sent every second. The message ID 7 refers to image frames messages sent at a specific frame rate. We used a frame rate equal to 20 fps at the drone side. Other message IDs refer to other types of messages (robot status, global motion, GPS info, acknowledgement).

It can be observed that video frames take a much longer time to be processed in the cloud as compared to other types of messages. The reason is due to the large size of the message carrying out image frames. Besides, the variation of the processing time is the highest for images. For an image, the minimum processing time is 0.73 ms, and the highest is 18.20 ms, with an average of 9.57 ms. For a heartbeat message, the minimum processing time is 0.24 ms, and the highest is 2.98 ms, with an average of 1.25 ms.

Overall, the average processing time at the cloud, including video frame streaming, is 7.25 ms (with computation offloading), whereas it drops down to 2.25 if we exclude video streaming (without computation offloading). As such, it is clear that computation offloading would induce three times more CPU cycles for forwarding image frames at the cloud side.

##### Average Delay

**Figure 10:**
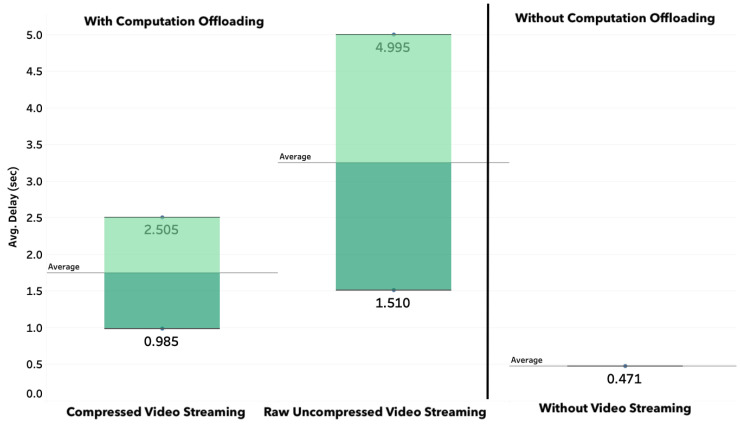
End-to-end network delays with and without computation offloading/video compression.

Figure 10 presents the average end-to-end delays with and without computation offloading. In the case of computation offloading, we consider the case of compressed video and uncompressed video. The results are presented for the case of ten drones streaming to the cloud with ten connected users through a Websocket. For statistical significance, the operation of drones was executed over 75 min for each case, and more than 90,000 packets in each case were considered for the average delay computation in Figure 10.

We observe that the average delay is less than 0.5 s when the video is processed locally (i.e., without computation offloading), and it reaches 3.2 s in case of computation offloading without video compression and 1.7 s with video compression. Naturally, the end-to-end delay values depend on the network conditions (during the experiments, the bandwidth is roughly 50 Mbps over the 4G connection), and also the image size used for streaming (824 KB in case of raw data, and 350 for the case of compressed data). However, the trends shown in Figure 10 illustrate the effect of computation offloading on the end-to-end delay and the fact that compression may help in achieving smoother real-time performance. The video stream quality can be made adaptive to the network condition based on the required frame-per-second and the maximum end-to-end delay requirements specific for every application.

**Figure 11:**
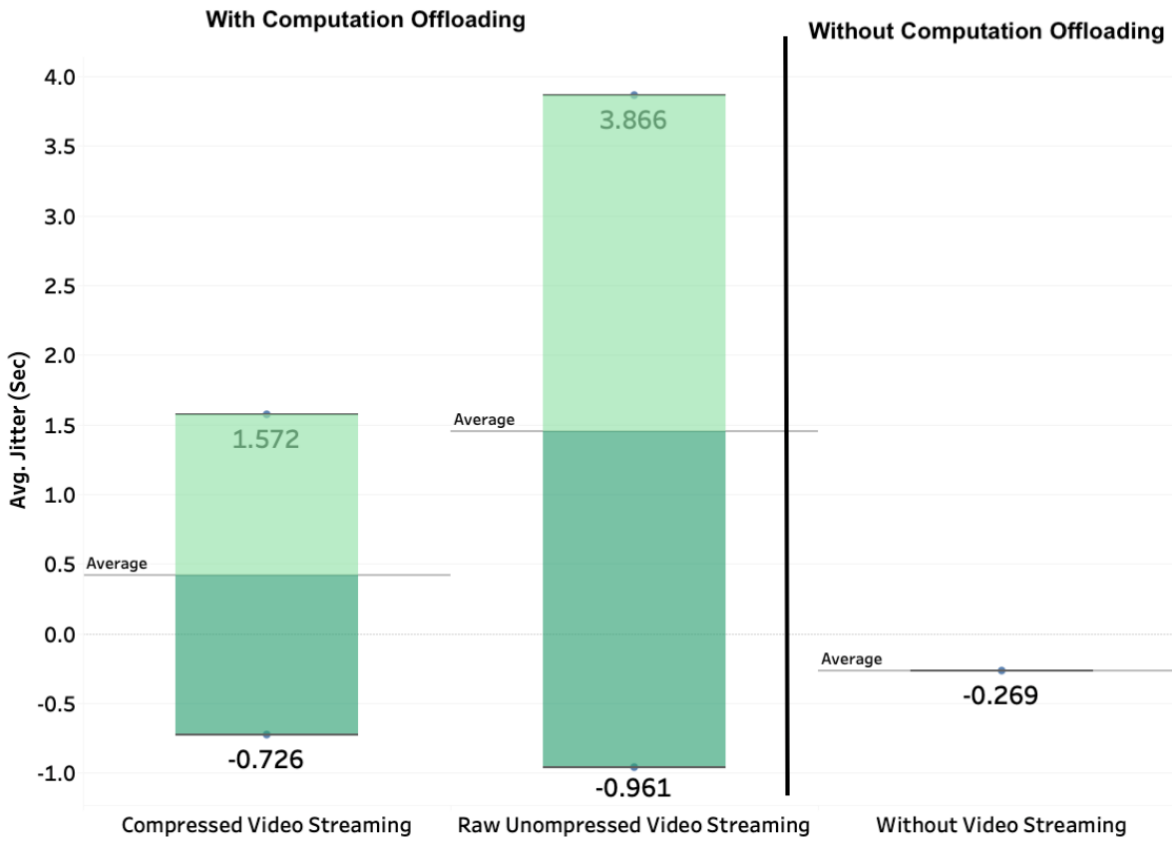
End-to-end network jitter with and without computation offloading/video compression.

We also analyze the impact of computation offloading and video compression on the network jitter. Figure 11 presents the end-to-end network jitter with and without computation offloading/video compression. The jitter is measured by the difference between the delays of two consecutive packets. The behavior of the jitter is similar to that of the delay in the sense that in the case of non-computation offloading, the jitter is as small as 0.563 s. With computation offloading, the jitter also depends on video compression being 1.2 s in the case of video compression and up to its double 2.4 s in the case of raw video streaming (without compression).

**Figure 12:**
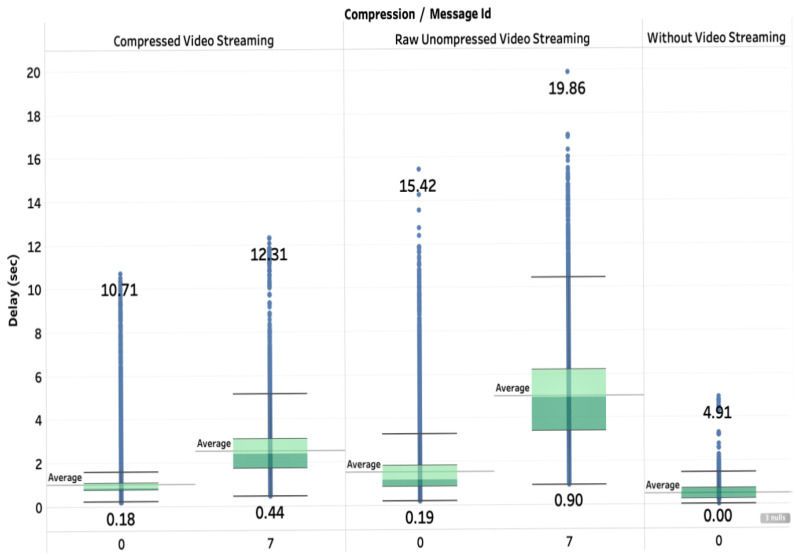
End-to-end network delays per message type.

This quantitative evaluation of the end-to-end delays and jitters illustrates the requirements in terms of bandwidth and latencies in the case of cloud-based deployment of deep learning applications for the Internet-of-Drones. The decision on whether or not to deploy the deep learning application onboard or in-cloud heavily depends on the available network resources to manage the additional communication cost between the drone and the cloud in what concerns video streaming. We analyze in more details the effect of video streaming in Figure 12.

Figure 12 presents the end-to-end network delays (in sec) for the DeepBrain system in the case of computation offloading per message type. In this figure, we consider the cases of message ID corresponding to a heartbeat message (0) and a message of type image (7). Furthermore, we conducted experiments with image compression and without image compression, to investigate the impact of the image size on the delay. In the case of image compression, we reduce the size of an image from 596 KB to 208 KB, meaning to 35% of the original size. We observe in Figure 12 that the compression mechanism allows to reduce the average end-to-end delay up to 56% for image messages, and 26% for heartbeat messages. Note that the end-to-end delays depend on the network condition at the time of the experiments, but the effect of compression is not dependent. We also observe that the delay of image message is longer than the delay of heartbeat message, as a result of the size difference. We also observe that current network technologies induce time jitter (i.e., delay variation), due to the best-effort network traffic offered service on the Internet and 4G connectivity. Using compression, the jitter was much reduced. Thus compression mechanisms can be an efficient alternative in improving the real-time quality of service of computation offloading of video streams to the cloud.

**Figure 13:**
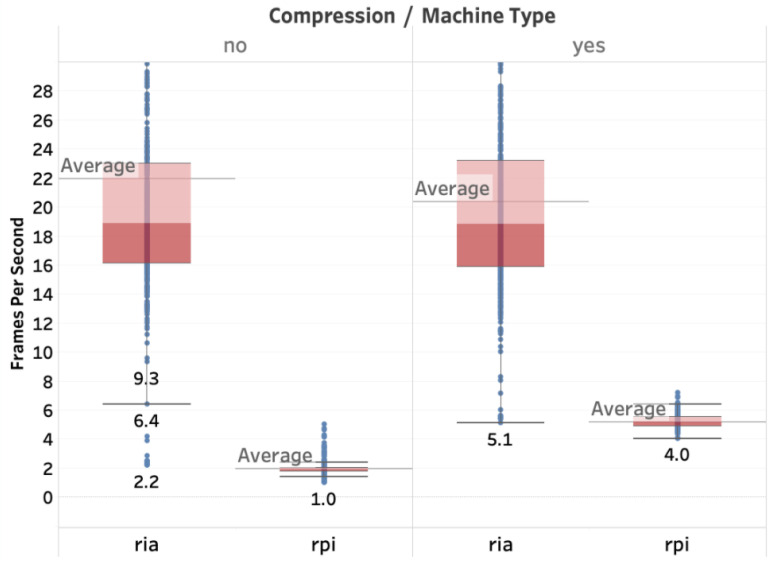
Frames per second (FPS).

Figure 13 depicts the number of frames per second (fps) received by the user application. We have measured the number of received frames per second by considering two cases. In the first case, the video is streamed from a Raspberry PI single-board computer, which is used as the autopilot of the drone with the Navio2 board. We refer to this case as RPI in Figure 13. In the second case, the video is streamed from a more powerful computer machine with a Core i5 processor and 8GB of RAM. We refer to this case as RIA in Figure 13, as it is the onboard computer of the Gaitech RIA E100 robot. The objective of testing two different broadcasting machines with different computation capabilities is to assess its impact on the streaming quality.

##### Frame Rate

Considering the impact of compression, we observe that using compression allows us to significantly increase the number of frames per second received from the end-user. In the case of the Raspberry PI streamer, the number of frames per second increased from 1 fps to 4 fps (four times more), whereas in the case of RIA, it slightly decreased from 22 fps to 20 fps (almost unchanged). In conclusion, the image compression much helps to improve the streaming quality of service in particular in low-capability devices.

However, while with a more powerful machine, it is possible to transmit frames at higher rates, the variation of the number of received frames at the end-user is also much more significant. Sending more frames induces additional delays and jitters in the network that impacts the regularity of reception of the image frames at the end-user. However, 50% of the frames are received at rates between 16 fps and 22 fps, which provides a decent quality-of-service with respect to the frame rate at the receiver.

### 4.3. Execution Times of Deep Learning Computing Tasks

In this section, we aim at evaluating the execution times of deep learning computing tasks on GPU-based platforms targeted for edge computing (connected to the drone) and on the cloud. The objective is to compare the execution times against their counterpart communication delays that we evaluated in the previous section. In the case of edge computing, we evaluated the inference time of object detection algorithms on two state-of-the-art boards used for inference at the edge, namely Jetson Nano and Raspberry Pi 4. For the case of cloud computation offloading, we measured the execution times of the same application on powerful GPUs, namely NVIDIA RTX 2080 Ti and NVIDIA GTX 1080. The specification of the devices for both edge and cloud is presented in Table 3.

The application that we used for comparing execution times is a Yolov4-tiny [45] object detection network, with input size 416 and half-precision floating-point format (FP16), pretrained on the COCO dataset [46]. We have tested it using a Tensorflow Lite (TFLite) framework, on a 1mn39s video (20 FPS) captured by a Hikvision camera, on different cloud-based and edge-based GPUs. It has to be noted that TFLite framework is required for the execution of deep learning applications on the edge, because the full Tensorflow framework is too heavily to be executed on the embedded devices, in contract to cloud-based devices. Table 4 shows the obtained results in terms of average execution time and average and standard deviation FPS. The execution time measures the full processing, including the frame reading using Python OpenCV library which takes a non-negligible time. The cloud-based GPUs show close performance (RTX 2080 Ti is only 8% faster) but are 12 to 15 times faster than Edge-based devices. The execution time on edge-based devices shows however much less variance. By contrasting the measured execution times against the communication delays, we can observe a balance between the computation offloading approach and the edge computing approach for the total communication and processing times. However, using computation offloading with some buffering to compensate jitters and delays allows us to leverage a much higher throughput than edge computing.

### 4.4. Lessons Learned

In the recent research works pertaining to the use of drones over the Internet, there is a major debate about whether the processing should be embedded at the edge level (i.e., in the drone side) [47,48,49] or it should be offloaded to the cloud [27,31,34]. Each approach has its own advantages and limitations in terms of cost, bandwidth requirement, processing speed, and energy consumption. In this paper, we contributed to this research area by conducting an experimental study that investigates the requirements and performance of Internet-of-Drones application with and without computation offloading, in terms of energy, real-time, frame rate and bandwidth. The results presented in this paper provide an insight on the tradeoff between using computation offloading and computation at the edge. We conclude that computation offloading is effective when sufficient network resources can be guaranteed for the video stream to be processed on the cloud and may allow a frame rate up to 20 frames per second. Nonetheless, for low-capability devices streaming at high frame rates might not be possible in this case, and compression has to be applied. The problem with compression is the loss of important features that could be crucial for deep learning models to extract objects and classify them. Thus, it could be more appropriate in such cases to process images at the edge with embedded GPU devices (e.g., Jetson Nano, Raspberry Pi) to be able to use high-resolution images that cannot be streamed through the Internet to the cloud due to lack of resources. Another strategy would also be to offload part of the computation to the cloud and part of it processed at the edge level. For example, some complex deep learning applications may consists of several levels of detections and classifications even for the same image. In such as case, it is possible to execute part of the computation on the edge (example a vehicle detection algorithm), and just send the cropped image of the detected object to the cloud to perform classification. This would save the communication cost because the cropped image would be much smaller that the original image collected by the drone. One limitation of our work is that we did not considered such a hybrid strategy in our experimental analysis. We are planning to address this in future works. Furthermore, the computation offloading with high-quality video may incur high end-to-end delays up to five seconds, which could be acceptable for delay-tolerant applications. However, the time saved in terms of communication in case of edge-based computation compared to cloud-based computation offloading will be lost in terms of processing because inference with the GPUs of embedded devices is much slower than the processing on cloud-based GPUs. According to our experimental results, it seems that the communication delay of images in cloud-based computation offloading might be a bit higher than the processing time of deep learning applications on edge. However, when using video stream buffering on the cloud side, it is possible to leverage the cloud GPUs’ full power to process the stream with high throughput reaching 12 frames/s (because streaming over the Internet may provide a frame rate up to 20 frames/s). In the case of edge computing on the drone’s side, the throughput will always be limited to an average of one frame per second. In conclusion, the adopted solution will depend on user requirements in terms of throughput.

## 5. Conclusions

This paper discusses the real time on-board processing problem of data collected by offloading computer vision activities from the drone to the cloud in order to reduce energy consumption due to heavy UAV calculation. The idea is to stream the UAV video into the cloud, which deploys a deep learning system for analyzing obtained data in real-time and proposes decisions for end-users. This work introduces the system architecture and addresses the technical challenges as well as the contribution to a car detection case via an experimental prototype. The results indicated that the system proposed is effective in reducing the energy consumption of UAVs while allowing computer vision applications to be performed in real time using CNN algorithms.

## Figures and Tables

**Table 1:** Summary of recent trends on computation offloading.

	Device Type	Problem/Approach	Deep Learning Applications	Validation	Main Result
**(Kang et al., 2017) [20]**	mobile	Analyze the calculation and data features of 8 Deep Neural Networks (DNN) architectures, Computer vision, speech, and processing applications for natural languages and demonstrate the balance between partitioning computation at many points within the network.	Yes	Experimental	Improves end-to-end latency, reduces mobile energy consumption and improves datacenter throughput.
**(Wamser et al., 2017) [21]**	drones	Demonstrate the effect of network condition on video streaming from drones over the cloud	No	Experimental	Improve the quality-of-service of the streaming over the cloud
**(Van Le et al., 2018) [17]**	adhoc mobile	reinforcement learning for offloading of ad-hoc mobile applications to the cloud using cellular networks	No	Simulation	Obtain optimal offloading
**(Tan et al., 2018) [22]**	mobile	Joint optimal connectivity, storage and computing resource management system for vehicular network using deep reinforcement learning approach	Yes	Simulation	Significant performance by optimum selection of parameters.
**(Wang et al., 2019) [23]**	mobile	Deep Reinforcement Learning techniques and Federated Learning framework with the mobile edge system	Yes	Simulation	Achieves near-optimal performance
**(Chaari et al., 2019) [24]**	robots	Kafka broker for offloading computer vision applications from robots to cloud	No	Experimental	Communication delays may increase execution times
**(Xu et al., 2019) [25]**	Mobile	offloading deep learning mobile applications of 5G networks	Yes	Simulation	Reduces delay for deep learning tasks
**(Qi et al., 2019) [26]**	vehicles	Deep reinforcement learning to obtain optimal offloading decisions	No	Simulation	online learning of computation offloading from vehicular services
**(Alelaiwi et al., 2019) [27]**	mobile	Deep-learning-based response-time prediction computation offloading method	Yes	Simulation	Reaches a Mean Absolute Percentage Error (MAPE) below 0.1 and an R-square greater than 0.6
**(Alam et al., 2019) [28]**	mobile	Deep Q-learning based code offloading method of computation in mobile edge/fog.	Yes	Simulation	The proposed offloading performs better for time and latency execution and energy consumption.
**(Ning et al., 2019) [29]**	mobile	Nonorthogonal Multiple Access (NOMA) system for mobile edge computing (MEC) vehicular network.	Yes	Simulation	Under the various network circumstances the scheme can increase transfer rate gain and offload efficiency.
**(Ning et al., 2020) [30]**	mobile	Deep-reinforcement-learning-based framework for 5G-enabled vehicle networks	Yes	Simulation	Achieved an overall better offloading cost.
**(Chen et al., 2020) [31]**	drones	Intelligent Task Offloading Algorithm (iTOA) for UAV edge computing network using a splitting Deep Neural Network (sDNN)	Yes	Simulation	Improves service latency performance by 33% and 60%, respectively.
**(Wu et al., 2020) [32]**	drones	Three-layer UAV-based Mobile Edge Computing (MEC) network architecture and the functions of task offloading and data communication are analyzed in IoT device layer, UAV based edge computing layer and MEC server layer	Yes	Simulation	The energy consumption of UAV is reduced, and the proposed algorithm is used to dynamically schedule the task offloading strategy.
**(Wang et al., 2020) [33]**	drones	Framework of task scheduling is presented in the unmanned aerial vehicle-aided mobile edge computing (UMEC)	Yes	Simulation	The implementation of the agent in computing tasks would reduce delays and energy consumption significantly.
**(Alioua et al., 2020) [34]**	drones	A new device architecture for offloading and exchanging computations. Then, a new device utility function is developed which combined calculation time, overhead energy, link quality, communications and computing costs	Yes	Experimental	More efficient time and energy average for data processing which ranges from 43 % to 97 % according to the calculation approach.
**DeepBrain**	drones	Design and develop a full-stack cloud-based architecture for computation offloading of deep learning applications in Internet-of-Drones	Yes	Experimental Performance Evaluation	Demonstrate the feasibility and performance of computation offloading of deep learning applications from drones connected through the Internet

**Table 2:** Energy consumption (non flying drone).

Scenarios	Voltage Decrease Rate (Volt Per Second)	Instant Power Consumption (Watt)
Computation Offloading	$1.159\times 10^{-4}$	3.2 Watt
Onboard GPU Processing	$2.284\times 10^{-4}$	6 Watt

**Table 3:** Specification of the cloud-based and edge-based devices used for evaluation of deep-learning algorithms.

	Device	CPU	GPU	RAM
Cloud-based devices	MSI Infinite workstation	Intel Core i9-9900K @ 3.7 GHz	RTX 2080 Ti (11 GB)	64 GB
	HP Omen workstation	Intel Core i7-8700K @ 3.7 GHz	GTX 1080 (8 GB)	64 GB
Edge-based devices for edge-computing	Jetson Nano	Quad-core ARM A57 @ 1.43 GHz	128-core Maxwell	4 GB
	Raspberry Pi 4	Quad core Cortex-A72 (ARM v8) @ 1.5GHz	Broadcom VideoCore VI	4 GB

**Table 4:** Average execution time, average frames per second (FPS) and its standard deviation for YOLOv4-tiny (input size 416) on different GPU types.

	GPU Type	Average Execution Time per Frame (s)	Average FPS	Standard Deviation
Cloud-based servers	RTX 2080 Ti	0.072	14.3	1.0
	GTX 1080	0.078	12.9	1.0
Edge-based devices for edge-computing	Jetson Nano	1.1	0.91	0.01
	Raspberry Pi 4	0.96	1.04	0.06
